# mGem: Opening Env and harnessing NK cell effector functions to eliminate HIV-1-infected cells

**DOI:** 10.1128/mbio.02807-25

**Published:** 2026-03-03

**Authors:** Jonathan Richard, Andrés Finzi

**Affiliations:** 1 Centre de Recherche du CHUM, Montreal, Quebec, Canada; 2 Département de Microbiologie, Infectiologie et Immunologie, Université de Montréal 5622, Montreal, Quebec, Canada; Albert Einstein College of Medicine, Bronx, New York, USA

**Keywords:** HIV-1, cure, CD4 mimetics, ADCC, non-neutralizing antibodies

## Abstract

Despite effective suppression of viremia by antiretroviral therapy, HIV-1 persists in long-lived cellular reservoirs. Novel approaches aimed at eliminating these reservoirs are therefore essential for an HIV-1 cure. Among emerging cure strategies, harnessing antibody-dependent cellular cytotoxicity (ADCC) has generated significant interest. In this mGem, we discuss how small CD4-mimetic compounds (CD4mc), by forcing envelope (Env) into more “open” conformations, thereby exposing conserved CD4-induced epitopes, can unlock the ADCC potential of non-neutralizing antibodies. We also highlight how type I interferons complement this approach by upregulating BST-2, thereby increasing Env at the cell surface, diminishing Vpu-mediated immune evasion, and enhancing NK cell effector functions. Together, these synergistic interventions provide a promising framework to improve immune recognition of infected cells and potentially reduce the size of the HIV-1 reservoir.

## PERSPECTIVE

## HIV-1 ADCC EVASION STRATEGIES

The advent of combination antiretroviral therapy (cART) has transformed HIV-1 infection into a manageable chronic condition by effectively suppressing viremia through the inhibition of multiple steps in the viral replication cycle (1, 2). However, cART alone cannot eliminate the virus. Integrated proviruses persist in long-lived cells, forming a stable latent reservoir capable of rapidly reinitiating viral replication upon treatment interruption (3–7). Consequently, the elimination of the viral reservoir remains the main obstacle to an HIV-1 cure.

Among the strategies being explored to overcome this challenge, approaches designed to exploit antibody-dependent cellular cytotoxicity (ADCC) have generated substantial interest. ADCC is an immune mechanism in which antibodies bound to viral antigens at the cell surface engage and activate immune effector cells, most notably, natural killer (NK) cells, leading to the elimination of the target cell (8). Being the only viral protein displayed on the surface of infected cells, the HIV-1 envelope (Env) trimer serves as the principal target for ADCC (9, 10). Although HIV-1 infection elicits a strong antibody response against Env, only very few people with HIV (PWH) develop antibodies with exceptional breadth, capable of neutralizing a wide range of circulating HIV-1 strains, and this happens only after several years of infection (11–15). These antibodies, termed broadly neutralizing antibodies (bnAbs), target epitopes naturally exposed on the native Env trimer present on both virions and infected cells. Accordingly, bnAbs targeting the CD4 binding site, the glycan-dependent V1/V2 loops, the V3 loops, the gp120-gp41 interface, and the membrane proximal region have been shown to mediate Fc effector functions against HIV-1-infected cells, such as ADCC, antibody-dependent cellular phagocytosis (ADCP), and antibody-dependent complement deposition (ADCD) (16–25). These functional properties led several clinical trials to evaluate whether bnAbs can control viremia and reduce the size of the HIV-1 reservoir (26).

Most naturally elicited antibodies in PWH exhibit little to no neutralizing activity and are collectively referred to as non-neutralizing antibodies (nnAbs) (27). Because they are abundant, naturally present in PWH and some recognize highly conserved epitopes, CD4-induced (CD4i) nnAbs represent an attractive alternative to bnAbs. CD4i epitopes are normally occluded within the native, “closed” Env but become exposed following Env engagement with CD4 (28–30). CD4 binds to the exterior gp120 subunit by inserting its phenylalanine 43 (Phe43) into a highly conserved pocket located at the interface of the inner and outer gp120 domains, called the Phe43 cavity (31). This interaction drives Env toward progressively more “open” conformations, transitioning from state 1, through the intermediate state 2, and ultimately reaching the fully open state 3 conformation (30, 32, 33). These structural rearrangements expose vulnerable CD4i epitopes targeted by some nnAbs (21, 22, 28–30, 34–39).

**Fig 1:**
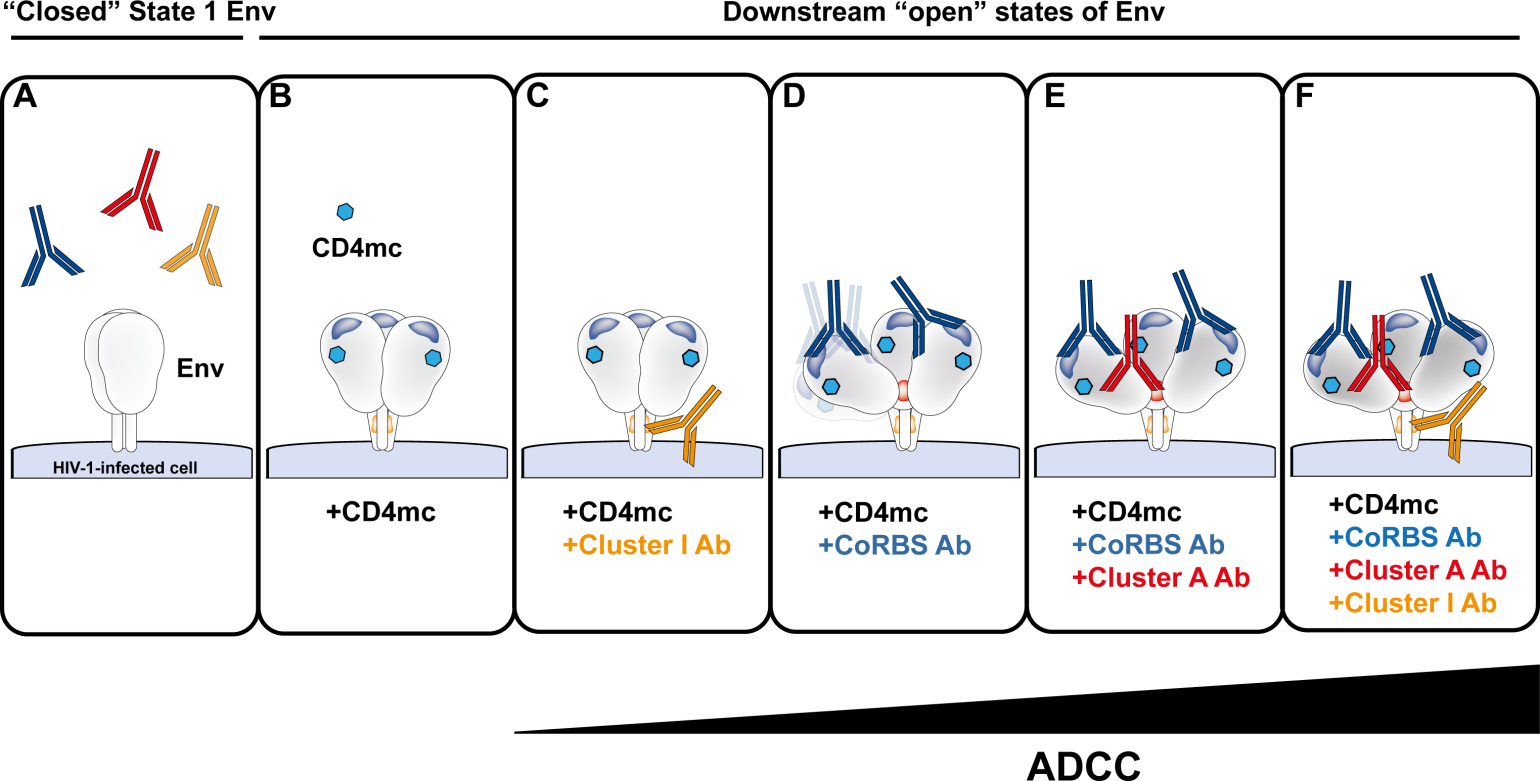
CD4mc expose vulnerable Env epitopes and enable nnAb-mediated ADCC. (**A**) Env expressed by primary HIV-1 isolates predominantly adopts a tightly “closed” conformation that is poorly recognized by nnAbs. (**B**) CD4mc shift Env toward downstream, more “open” conformations, thus exposing the gp41-cluster I region (orange) and the coreceptor binding site (CoRBS, dark blue). (**C**) This enables anti-cluster I Abs to bind and mediate ADCC. (**D**) Anti-CoRBS antibodies further “open” the trimer and expose the gp120 cluster A epitope (red), stabilizing more “open”, asymmetric Env conformations that position antibodies optimally for Fc receptor engagement and ADCC. (**E**) Binding of anti-cluster A antibodies further enhances ADCC and stabilizes the “open” State-2A Env conformation. (**F**) Addition of anti-cluster I to the anti-CoRBS Abs/anti-cluster A Abs/CD4mc maximizes ADCC responses.

While CD4i nnAbs are elicited early during natural infection, persist over time, and possess strong ADCC potential (20, 28, 40–42), HIV-1 has evolved multiple strategies to minimize their ability to recognize infected cells. The selective pressure imposed by anti-Env Abs likely contributed to the evolution and conservation of mechanisms that limit exposure of CD4i Env epitopes (43). One such strategy is the propensity of primary HIV-1 isolates to maintain Env in a predominantly “closed” state 1 conformation that is intrinsically resistant to nnAbs (18, 21, 22, 30, 34, 44). This closed architecture is stabilized through numerous intra-trimer interactions involving the V1, V2, and V3 loops, as well as the gp120 β20–β21 region (32, 45, 46). Disruption of these contacts shifts Env toward more “open” conformations and increases the susceptibility of infected cells to nnAb-mediated ADCC (36). Another major evasion mechanism relies on the ability of HIV-1 to reduce Env-CD4 interactions by actively removing CD4 from the surface of infected cells. This CD4 downregulation, mediated by the accessory proteins Nef and Vpu, as well as Env itself, is highly conserved among primary isolates (28, 29, 38, 39). Importantly, this occurs before Env reaches the cell surface, thereby preventing premature exposure of vulnerable CD4i epitopes (21). As a result, the Env trimers present on infected cells remain largely invisible to recognition by naturally elicited nnAbs in PWH, thereby protecting these cells from ADCC (Fig. 1A) (28). Consistent with this, CD4 accumulation due to deletion of Vpu and/or Nef or removal of the Env cytoplasmic tail is sufficient to “open up” Env, expose its vulnerable epitopes, and render the cells susceptible to ADCC by nnAbs (20, 22, 28, 34, 38, 39).

## UNLOCKING nnAb ADCC POTENTIAL USING CD4mc

Given the inherent vulnerability of “open” Env to nnAbs, considerable progress has been made in exploiting small CD4-mimetic compounds (CD4mc) to force Env to sample downstream “open” conformations, thereby unlocking the ADCC potential of nnAbs. Several classes of CD4mc, differing in potency and breadth, have been developed and recently reviewed in references 47, 48. By binding within the gp120 Phe43 cavity, CD4mc induce conformational rearrangements that expose epitopes recognized by CD4i Abs (Fig. 1). This renders HIV-1-infected CD4^+^ T cells susceptible to ADCC mediated by these Abs, which are abundant in biological fluids from PWH, including plasma, breast milk, and cervicovaginal lavage (49). While CD4i ADCC-mediating Abs have also been detected in semen (50), it remains unknown whether CD4mc can similarly enhance ADCC activity in this compartment. This strategy has been shown to sensitize *ex vivo*-expanded CD4^+^ T cells from PWH on cART to ADCC mediated by autologous plasma and effector cells (49) and to contribute to the elimination of HIV-1 reservoir cells *in vivo* in humanized mice (51). Recent findings demonstrate that CD4mc can render infected cells susceptible to ADCC by plasma collected as early as 3 weeks post-estimated date of infection (EDDI), highlighting their potential for early intervention (40). Remarkably, the activity of CD4mc is not restricted to CD4^+^ T cells; they can also sensitize HIV-1-infected macrophages to ADCC mediated by nnAbs and plasma from PWH (39, 52). Similarly, eCD4Ig, a fusion protein of CD4 domains 1 and 2, an antibody Fc domain, and a short tyrosine-sulfated peptide, was shown to promote Env opening, thereby exposing CD4i Env epitopes on HIV-1-infected cells (53). Accordingly, eCD4Ig can mediate ADCC directly through its Fc domain but also enhance ADCC mediated by nnAbs and sera from PWH (53).

Multiple families of CD4i nnAbs contribute to plasma-mediated ADCC in the presence of CD4mc. These include antibodies targeting the gp41 cluster I region, the coreceptor binding site (CoRBS), and the cluster A region (20, 22, 41, 52, 54, 55). The levels of these three families of nnAbs positively correlate with plasma-mediated ADCC activity (40, 41). CD4mc induce conformational changes that resemble, but do not fully replicate, those triggered by membrane-bound CD4. Like CD4, CD4mc directly expose the gp41 cluster I region, thus sensitizing infected cells to ADCC mediated by monoclonal antibodies (mAbs) targeting this conserved epitope (Fig. 1C) (20, 22, 52). Anti-cluster I Abs are particularly important early after infection, appearing as soon as 3 weeks post-EDDI, before anti-CoRBS and anti-cluster A Abs, and thus contribute more substantially to plasma ADCC activity at this stage of infection (40). Consistent with their functional relevance, administration of the anti-cluster I mAbs 246D, in combination with CD4mc, was shown to significantly reduce HIV-1 replication in humanized mice (20).

Although CD4mc broadly expose the CoRBS on Env, anti-CoRBS binding does not always translate into ADCC. These antibodies were initially considered poor ADCC mediators (22, 56–58). The recently developed and more potent indoline CD4mc CJF-III-288, however, uniquely sensitizes HIV-1-infected cells to anti-CoRBS-mediated ADCC (54). This feature contributes to its enhanced ADCC activity. Indeed, by harnessing the full potential of CD4i nnAbs, CJF-III-288 more efficiently sensitizes *ex vivo* and *in vitro* HIV-1-infected cells to ADCC mediated by PWH plasma (54). Remarkably, unlike previous CD4mc, administration of CJF-III-288 with the anti-CoRBS mAbs 17b is sufficient to significantly delay viral rebound in infected humanized mice, highlighting its therapeutic potential (54). Mechanistically, CJF-III-288, together with anti-CoRBS Abs, promotes a more “open,” asymmetric Env conformation that positions anti-CoRBS in orientations favorable for Fc receptor engagement (54). Structural analysis shows that the bound anti-CoRBS Abs exhibit greater conformational flexibility, permitting a wider array of Fab and Env trimer orientations that likely promote more effective Fc receptor interactions (54) (Fig. 1D).

Unlike membrane-bound CD4, CD4mc cannot expose on their own the highly conserved gp120 cluster A region. Instead, CD4mc rely on anti-CoRBS Abs to further open the Env trimer, finally allowing binding of anti-cluster A Abs (57). This combination of nnAbs and CD4mc has been shown to mediate potent ADCC against cells infected with diverse HIV-1 strains (52, 54, 57, 59), with the Fc portion of both nnAbs contributing to this response (58). This nnAb/CD4mc cocktail stabilizes state 2A, which is not on the pathway required for Env-mediated fusion (59). In HIV-1-infected humanized mice, administration of a cocktail comprising a CD4mc and anti-CoRBS/anti-cluster A Abs led to substantial reductions in the viral reservoir across multiple tissues and significantly delayed viral rebound after ART cessation, reflecting a measurable decrease of the functional HIV-1 reservoir (51).

Recent addition of an anti-gp41 cluster I mAb has further enhanced the potency and breadth of this cocktail (52). Its effectiveness correlates with a pronounced disruption of the native “closed” Env conformation (state 1) and stabilization of downstream, more “open” Env states (52). By simultaneously targeting four conserved regions of Env (Phe43 cavity, CoRBS, cluster A, gp41 cluster I), the cocktail demonstrates substantial therapeutic potential, surpassing several broadly neutralizing antibodies (bnAbs) in recognizing and mediating ADCC against *ex vivo*-expanded CD4^+^ T cells from ART-treated PWH (52). Further preclinical evaluation will be essential to determine the efficacy of this improved cocktail in reducing the HIV-1 reservoir. Due to its breadth, this cocktail is currently being used to detect and characterize Env-expressing reservoir cells (60).

It has been suggested that selective pressure from ADCC-mediating antibodies can drive the emergence of immune escape variants (61). However, these findings were largely based on experimental systems using target cells coated with Env peptides, leaving it unclear whether a similar escape occurs in the context of biologically relevant target cells expressing native, trimeric Env. Although we cannot entirely exclude the possibility that a CD4mc/nnAb cocktail could select for escape variants, such mutations have not been detected among rebounding viruses in humanized mice following CD4mc/nnAb administration (51). Of note, CD4mc/nnAb combinations target highly conserved epitopes that are critical for viral entry and fitness (52), thus raising the bar for viral escape.

To date, the efficacy of CD4mc/nnAb combinations in eliminating HIV-1-infected cells and reducing the viral reservoir has been evaluated primarily *in vitro* and *ex vivo*, but also *in vivo* in humanized mice. In this context, transgenic and knock-in humanized mice expressing human interleukin-15 (IL-15) have been employed to support NK cell development and Fc-mediated effector functions (20, 51, 54). Although these preclinical models do not fully recapitulate the human immune system, they have been shown to sustain robust development of both circulating and tissue-resident NK cell populations (62). Notably, CD56^dim^CD16^+^ NK cells, the subset mediating ADCC, were present at frequencies comparable to those observed in humans, displayed similar phenotypic diversity, and were capable of mediating ADCC both *in vitro* and *in vivo* (62, 63).

To evaluate the potential translation of these findings into human, recent studies evaluated the safety, pharmacokinetics, and biological activity of an indane-based CD4mc (BNM-III-17) in SHIV-AD8-EO-infected rhesus macaques (64). While this study established a well-tolerated dosing regimen and demonstrated the feasibility of large-scale CD4mc production for NHP studies, treatment did not result in a significant reduction in plasma viral load. This limited efficacy likely reflects both the relatively low CD4mc concentrations administered and the low titers of naturally elicited CD4i nnAbs in these animals. Future NHP studies should therefore evaluate the coadministration of higher concentrations of the potent CD4mc CJF-III-288 with an optimized cocktail of CD4i nnAbs (targeting cluster A, CoRBS, and cluster I epitopes) and complementary strategies to harness NK cell-mediated effector functions, in order to maximize clearance of the HIV-1 reservoir.

## ENHANCING THE SUSCEPTIBILITY OF HIV-1-INFECTED CELLS AND IMPROVING NK CELL EFFECTOR FUNCTIONS USING TYPE I IFN

**Fig 2:**
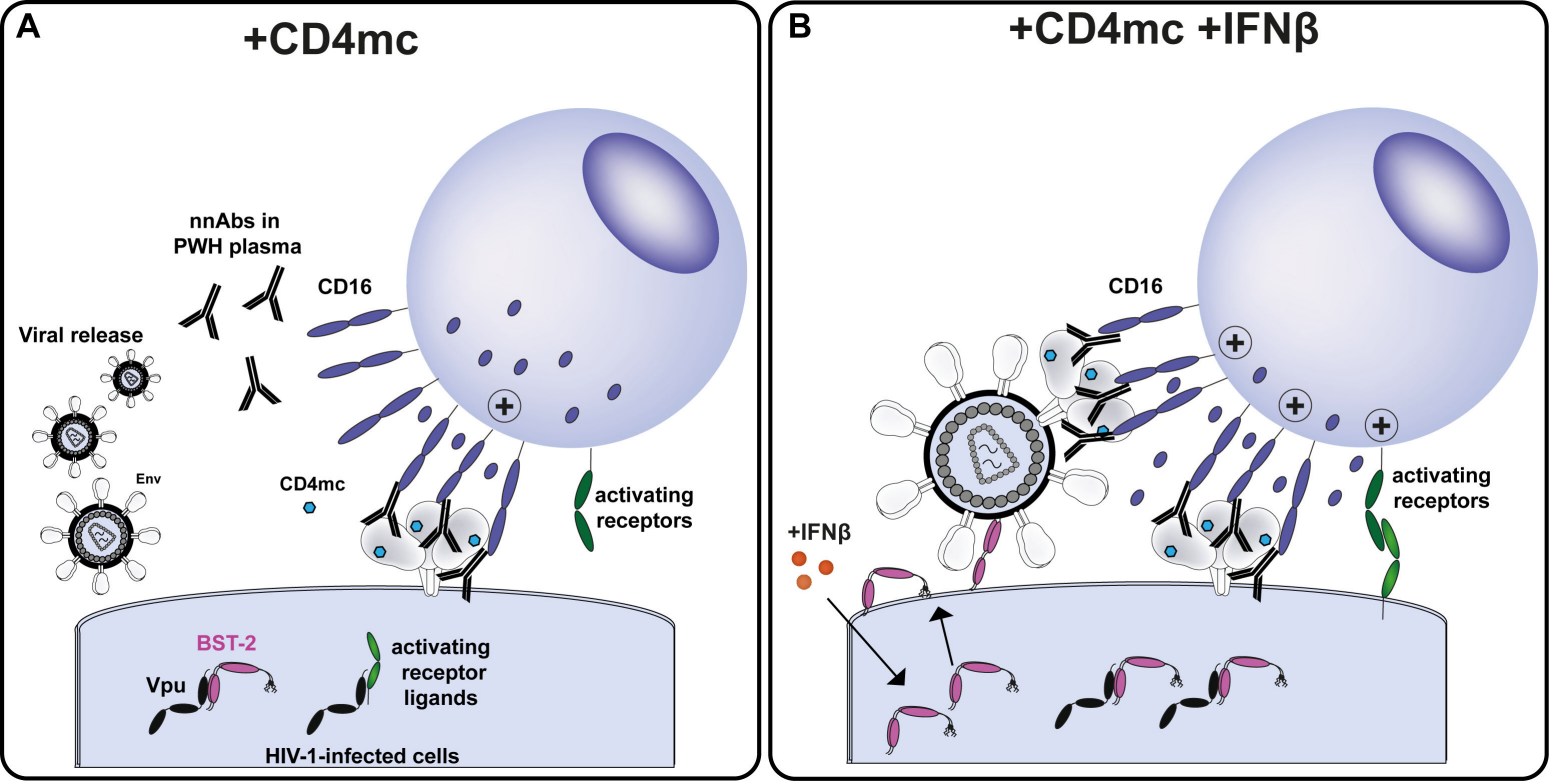
Type I IFN upregulates BST-2, increases Env levels at the cell surface, and limits Vpu’s ability to evade NK cell effector functions. (**A**) CD4-mimetic compounds (CD4mc) force Env into more “open” conformations, sensitizing HIV-1-infected cells to ADCC mediated by non-neutralizing antibodies present in plasma from PWH. However, this response is dampened by Vpu, which counteracts BST-2 to promote virion release and prevent surface Env accumulation. Vpu also downmodulates several activating NK cell ligands, which further reduce NK cell responsiveness. (**B**) Treatment with type I IFN markedly upregulates surface BST-2 even in the presence of Vpu. This results in virion tethering and increased Env retention at the cell surface, expanding the pool of Env accessible to CD4mc. IFN-induced BST-2 upregulation also affects Vpu’s polyfunctionality: Vpu preferentially targets BST-2 over NK cell ligands, ultimately promoting stronger NK cell activation.

It is well established that HIV-1 also evades ADCC by tightly controlling surface Env expression through Vpu-mediated antagonism of BST-2 (Tetherin) (20, 29, 65, 66). Viral release mediated by Vpu counteraction of BST-2 reduces Env accumulation at the cell surface, thereby limiting recognition by ADCC-mediating antibodies (20, 29, 65–67) (Fig. 2A). Therefore, the type I IFN responsiveness of BST-2 could be exploited to overcome this immune evasion mechanism. IFN treatment significantly upregulates BST-2 at the cell surface, despite Vpu expression, resulting in virion tethering and increased Env exposure (65, 68). This enhances recognition of infected cells by bnAbs (65) and increases the pool of Env accessible to CD4mc, thereby strengthening CD4mc-mediated exposure of CD4i epitopes and boosting ADCC mediated by plasma from PWH (68) (Fig. 2B).

Vpu also promotes NK cell evasion by downregulating several ligands of NK cell-activating/coactivating receptors—including NTB-A, DNAM-1, and 2B4—from the surface of HIV-1-infected cells (69–71). This downregulation impairs both direct NK cell cytotoxicity and ADCC, as these receptors synergize with CD16 to stimulate NK cell effector functions (69–72). BST-2 upregulation following type I IFN treatment not only increases surface Env levels but also diminishes Vpu polyfunctionality, thereby reducing its ability to simultaneously antagonize multiple substrates (72) (Fig. 2B). Emerging evidence supports a hierarchy of Vpu substrates under IFN-induced conditions: when BST-2 is upregulated, Vpu preferentially targets BST-2 over its other substrates (69, 72, 73), leading to the accumulation of NK cell ligands at the cell surface and enhanced NK cell activation (69, 72). Furthermore, IFN can directly boost NK cell effector functions, increasing their ability to recognize and eliminate HIV-1-infected cells (74, 75). Together, these findings highlight the potential of leveraging type I IFN to potentiate the activity of nnAb/CD4mc combinations.

However, type I IFNs have a paradoxical role in HIV-1 infection, exerting both antiviral and immunopathological effects. Chronic or systemic IFN signaling has been associated with immune activation and exhaustion, as well as variable efficacy in reducing the viral reservoir in preclinical models (76, 77). Nevertheless, short-term administration of IFNα2a/b to ART-suppressed PWH has been reported to be safe and associated with increased NK cell activation, plasma HIV control, and integrated HIV DNA decrease (78–80). Importantly, IFNα subtypes differ markedly in their biological activity despite signaling through the same receptor, with certain subtypes displaying enhanced antiviral potency and more favorable immunomodulatory profiles than the clinically used IFNα2a/b (72, 81–83). In this context, a transient and carefully timed, subtype-selective IFN intervention may potentiate nnAb/CD4mc-mediated ADCC.

## CONCLUSIONS AND PERSPECTIVES

Modulating Env conformation with CD4mc represents a promising strategy to unlock the potential of CD4i nnAbs to eliminate HIV-1-infected cells via ADCC. Whether CD4mc-mediated Env opening can also sensitize infected cells to additional Fc-dependent effector mechanisms, such as ADCP or ADCD, remains to be determined. A major challenge for antibody-based reservoir clearance strategies is the anatomical distribution of effector cells. The frequency of CD56^dim^CD16^+^ NK cells is reduced in lymphoid tissues relative to peripheral blood, despite these tissues harboring the majority of the latent HIV reservoir (84–87). Although monocytes and other myeloid cells within lymphoid compartments may also contribute to Fc-mediated clearance, strategies aimed at redirecting and empowering CD16^+^ NK cells in lymphoid tissues could substantially improve the therapeutic efficacy of immunotherapies (88, 89).

Several avenues exist to further enhance the potency of CD4mc/nnAb-based interventions. Fc engineering, including point mutations and Fc afucosylation, has been shown to markedly increase ADCC activity (90–94). Additionally, IgG3 variants of nnAbs may offer advantages over the IgG1 isotypes currently used, owing to their substantially longer and more flexible hinge region, which can improve epitope accessibility and Fc receptor engagement (95–97).

Finally, type I IFN also emerges as an attractive complementary approach. By increasing surface Env accumulation and directly enhancing NK cell effector functions, IFN treatment may significantly strengthen the capacity of CD4mc/nnAb cocktails to eliminate HIV-1-infected cells.
